# Sequential delivery of dual drugs with nanostructured lipid carriers for improving synergistic tumor treatment effect

**DOI:** 10.1080/10717544.2020.1785581

**Published:** 2020-07-02

**Authors:** Man Xu, Guangmeng Li, Haoxiang Zhang, Xiaoming Chen, Yi Li, Qianming Yao, Maobin Xie

**Affiliations:** a Qingyuan People’s Hospital, The Sixth Affiliated Hospital of Guangzhou Medical University, Qingyuan, China; b Department of Biomedical Engineering, School of Basic Medical Sciences, Guangzhou Medical University, Guangzhou, China; c School of Materials, The University of Manchester, Manchester, UK

**Keywords:** Temozolomide, curcumin, synergistic treatment, nanostructured lipid carriers, sequential drug release

## Abstract

To improve synergistic anticancer efficacy and minimize the adverse effects of chemotherapeutic drugs, temozolomide (TMZ) and curcumin (CUR) co-loaded nanostructured lipid carriers (NLCs) were prepared by microemulsion in this study. And the physicochemical properties, drug release behavior, intracellular uptake efficiency, *in vitro* and *in vivo* anticancer effects of TMZ/CUR-NLCs were evaluated. TMZ/CUR-NLCs showed enhanced inhibitory effects on glioma cells compared to single drug loaded NLCs, which may be owing to that the quickly released CUR can sensitize the cancer cells to TMZ. The inhibitory mechanism is a combination of S phase cell cycle arrest associated with induced apoptosis. Notably, TMZ/CUR-NLCs can accumulate at brain and tumor sites effectively and perform a significant synergistic anticancer effect *in vivo*. More importantly, the toxic effects of TMZ/CUR-NLCs on major organs and normal cells at the same therapeutic dosage were not observed. In conclusion, NLCs are promising nanocarriers for delivering dual chemotherapeutic drugs sequentially, showing potentials in the synergistic treatment of tumors while reducing adverse effects both *in vitro* and *in vivo*.

## 1. Introduction

Gliomas are rarely curable since they are frequently involved with normal brain tissue (Krex et al., 2007). Surgical resection followed by chemotherapy and radiotherapy, are standard treatment options (Ganipineni et al., 2018; Lee et al., 2018). However, post treatment survival remains only 12–15 months (Cloughesy et al., 2014). Tumor resistance and the blood-brain barrier (BBB) are the main impediments, which make traditional chemotherapy drugs (such as temozolomide, TMZ) ineffective. BBB is a physiological barrier between brain and blood (He et al., 2017; Yao et al., 2018), which is mainly composed of brain capillary endothelial cells that can prevent 98% of drugs from accessing the brain (Karim et al., 2016). Previous studies showed that lipid nanocarriers with particle size around 20–200 nm could avoid renal glomerular filtration and easily pass through BBB (Soares et al., 2018; Jo et al., 2015; Sachdeva et al., 2015).

Over the past decade, nanoparticle-based delivery systems have been extensively explored in biomedical fields for prevention, diagnosis and treatment of diseases (Yu et al., 2019a; Sun et al., 2019a; Hu et al., 2018). The nanoparticle systems have unique properties which are able to increase drug solubility, enhance drug targeting, modulate release profile, load and deliver multiple drugs simultaneously (Chen et al., 2018a; Yu et al., 2019b; Pu et al., 2019). Nanostructured lipid carriers (NLCs) are promising drug delivery systems. NLCs are mainly composed of solid and liquid lipids and have three types: disordered, amorphous and multiple (Carvajal-Vidal et al., 2019; Li et al., 2017). NLCs can increase the solubility of insoluble drugs, reduce their toxic effects and prolong their residence time in glioma cells (Soares et al., 2018). Compared with solid lipid nanoparticles, NLCs have higher drug loading and encapsulation efficiency (EE) (Pastor et al., 2019). Indeed, NLCs’ small particle size (< 100 nm) and their intrinsic properties (such as lipid property and good biocompatibility) could facilitate crossing BBB (Meng et al., 2016). Therefore, NLCs are potential candidates for treating central nervous system diseases. However, overcoming tumor resistance remains another challenge.

TMZ is an imidazotetrazine alkylating agent for treating gliomas (Song et al., 2016) in clinical. Its active product, 5-(3-methyltriazen-1-yl) imidazole-4-carboxamide (MTIC), exerts cytotoxic effects through the mismatch repair of methylated adducts (Stupp et al., 2017; Han et al., 2018; Acerbi et al., 2018). Recent study showed that functionalized with folic acid (FA), the triblock polymer coated magnetic nanoparticles loaded with TMZ were able to cross BBB, improving the therapeutic efficiency on glioma (Afzalipour et al., 2019). In addition, the co-loaded paclitaxel (PTX) and TMZ hydrogel showed synergistic antitumor effects on glioma (Zhao et al., 2019). TMZ-NLCs have shown inhibitory effect on glioblastomas (Pastor et al., 2019). Nevertheless, the anticancer effect of TMZ-NLCs was significantly reduced due to tumor resistance (Zhang et al., 2010). Curcumin (CUR) is a natural compound extracted from the rhizome of ginger plants (Xie et al., 2015a). CUR exerts an anticancer effect by inducing differentiation among malignant tumor cells, promoting apoptosis and inhibiting growth in various cell cycle stages. Importantly, CUR has shown low cytotoxicity on normal cells (Kunwar et al. 2008). In our previous studies, supercritical-based nanoCUR revealed higher solubility and bioavailability than raw CUR, showing enhanced antibacterial and anticancer properties (Xie et al., 2015b; Xie et al., 2016a; Xie et al., 2016b). It was also reported that CUR-NLCs could increase the brain tumor-targeting efficiency of encapsulated CUR, which ultimately increased inhibitory efficiency of encapsulated CUR from 19.5 to 82.3% (Chen et al., 2016). Combination of CUR and TMZ showed a synergistic effect on generation of reactive oxygen species (ROS), which enhanced the sensitivity glioma cells to TMZ (Yin et al., 2014). Moreover, combination of CUR and TMZ inhibited the activities of phosphorylated AKT and mTOR significantly, implying that CUR can be used to increase the inhibitory effect of TMZ on glioma to overcome tumor resistance (Yin et al., 2014).

Therefore, TMZ/CUR-NLCs were prepared to improve the anticancer effects and minimize adverse effects in this study, and their physicochemical properties were characterized. Then, the drug release behavior and intracellular uptake ability were evaluated, followed by the *in vitro* anticancer effect and cytotoxicity evaluation. Finally, the *in vivo* biodistribution, toxicity, and synergistic anticancer effects of TMZ/CUR-NLCs were studied, and a proposed mechanism was revealed.

## 2. Material and methods

### 2.1. Materials and cell lines

Glyceryl monostearate, medium chain triglycerides and poloxamer 188 were purchased from AIKE REAGENT Co., Ltd. (Chengdu, China). Absolute ethanol was purchased from the DAMAO Chemical Reagent Factory (Tianjin, China). Phosphoric acid and methanol were supplied by FUYU CHEMICAL (Tianjin, China). TMZ and CUR were purchased from Aladdin Co., Ltd. (Shanghai, China). 3-(4,5-Dimethylthiazol-2-yl)-2,5-diphenyltetrazolium bromide (MTT) was purchased from Med Chem Express Co., Ltd. (New Jersey, USA). Hoechst 33342 Live Cell Staining Solution was obtained from Beyotime Co., Ltd. (Shanghai, China). All other chemical compounds were of analytical purity. Rat glioma cell line (C6) and human brain glial normal cell line (HEB) were obtained from Jennio Biotech Co., Ltd. (Guangzhou, China).

### 2.2. Preparation of NLCs

Blank and drug loaded NLCs were prepared by the microemulsion method with minor modifications according to the previous report (Teixeira et al., 2017). The solid lipid and the liquid lipid were uniformly mixed at a ratio of 7:3 and heated to 83 °C; then, CUR and TMZ were added separately into the mixed solution until the ratio of TMZ/CUR/lipid was 2:1:40 (weight/weight/weight). Afterwards, poloxamer 188 (9%, weight/weight) and ethanol (9%, weight/weight) were uniformly mixed with 8 mL of deionized water and was heated to 83 °C with plastic wrap. The aqueous phase of mixed surfactant was added into the liquid lipids, and the resulting nanoemulsions were formed by magnetic stirring at a speed of 500 rpm and a constant temperature (83 °C) for 20 min. The nanoemulsions were then quickly immersed into ice water (0-4 °C) and magnetically stirred for 4 h at a speed of 500 rpm, forming the drug loaded NLCs. The prepared drug loaded NLCs were purified by freeze-drying (SCIENTZ-10N, XINZHI, China) for 48 h to obtained dry samples for the next characterizations. The other groups were prepared in the same way. All dry samples were stored in dark and dry environment at room temperature.

### 2.3. Morphological and physical characterizations

A small amount of NLCs was dispersed evenly on a copper mesh. Then, 2% of phosphotungstic acid was added onto the copper wire dropwisely. After drying, the copper mesh was removed. The obtained morphology and inner structure of NLCs were observed under FEI Tecnai G2 F20 S-TWIN transmission electron microscope (TEM), (FEI, USA). A small amount of NLC solution was mixed with distilled water. The Malvern laser particle size analyzer was used to measure particle size, size distribution, polydispersity index (PDI) and zeta potential of blank NLCs and drug loaded NLCs.

### 2.4. Determination of encapsulation efficiency

EE was measured by ultrafiltration method. In details, the obtained NLC powders were diluted with absolute ethanol and mixed ultrasonically, then placed in 1 mL centrifuge tubes, and centrifuged at 11,000 rpm for 15 min. The supernatant was tested for the quantification of total loaded TMZ and CUR. The total amount of CUR and TMZ in the lipid phase was determined by an ultraviolet (UV)-visible spectrophotometer (UV-2600, SHIMADZU, Japan) at 420 nm and 324 nm, respectively. To remove free drugs attached to the surface, NLCs were dispersed in purified water in filter device of ultrafiltration centrifuge tube (10,000 MWCO) and centrifuged at 10,000 rpm for 30 min. Then, the liquid in centrifuge tube was taken out and diluted with methanol. The content of CUR and TMZ in the NLCs layer was determined by UV-visible spectrophotometer at 420 nm and 324 nm, respectively. The EE of drugs can be calculated using the following Equation (1).

(1) $$\text{Encapsulation}\;\text{efficiency}\;(\%)=\frac{W_{\mathrm{total}}-W_{\mathrm{free}}}{W_{\mathrm{total}}}\times 100\%$$

### 2.5. Differential scanning calorimetry

The thermal properties of NLCs were studied by differential scanning calorimetry (DSC) (DSC 214, NETZSCH, Germany). Free drug, blank-NLCs, TMZ-NLCs, CUR-NLCs and TMZ/CUR-NLCs were examined. 5 mg of samples were placed into an aluminum crucible. Sample disks were then placed on the sample holder of the DSC tank. An empty sample tray was used as a reference. The experiment began after the baseline was stabilized and temperature programed. The nitrogen flow rate was 250 mL min^−1^. The temperature range was set from 25 °C to 300 °C, and the heating rate was set to 10 °C min^−1^.

### 2.6. In vitro drug release studies

The *in vitro* cumulative release of TMZ and CUR from NLCs were measured using an dialysis method in phosphate buffered saline (PBS) at pH 7.2. 15% (v/v) of ethanol in PBS was to facilitate CUR detection (Fang et al., 2012). The dialysis bag (with a molecular weight cutoff of 8000) was used to retain the NLCs and allow the released drugs to disperse into the media. In details, 20 mg of lyophilized products were added to 1 mL of PBS. The resulting suspension was poured into the dialysis bag, which was then tightly bound at both ends and placed in a 4 mL centrifuge tube containing 3 mL of PBS. Then, the entire system was placed on a magnetic stirrer at a speed of 80 rpm at 37 °C. At given time intervals, 1 mL of the surrounding medium was withdrawn for the analysis of released TMZ and CUR by a UV-visible spectrophotometer at 324 nm and 420 nm, respectively. Immediately following the withdrawal, the tube was compensated by adding 1 mL of fresh PBS solution to maintain a constant volume. The same amount of free drugs dissolved in PBS were measured as control.

### 2.7. Cell culture and proliferation

Cells (C6 and HEB) were maintained in Roswell Park Memorial Institute (RPMI) 1640 medium (Thermo Fisher Scientific, Shanghai, China) supplemented with 10% FBS (Thermo Fisher Scientific, Shanghai, China) and 1% pen-strep (100 U mL^−1^ penicillin and 100 mg mL^−1^ streptomycin) (Thermo Fisher Scientific, Shanghai, China) in a 37 ± 0.2 °C humidified incubator containing 5% CO_2_. Cells were cultured into a monolayer and counted using an Auto T4 Cellometer (Nexcelo Bioscience LLC, Massachusetts, USA).

### 2.8. Cellular uptake study

Cellular uptake efficiency of TMZ/CUR-NLCs (2:1) and TMZ/CUR (control) was evaluated by the fluorescence images observed in treated cancer cell line C6. A confocal petri dish was seeded with 5 × 10^4^ cells (1 × 10^4^ cm^−2^) and incubated overnight to allow the cells to attach to dish. TMZ/CUR-NLCs (2:1) and control were then added to co-culture with cancer cells for 0.5 h and 4 h, respectively. The total drug concentrations were 10 μg mL^−1^. Thereafter, the treated cancer cells were washed with 1 × PBS twice, and nuclei were stained with 10 μL of Hoechst 33342 Live Cell Staining Solution for 30 min. Stained cells were observed using a Leica SP8 confocal laser scanning microscope (Leica, Weztlar, Germany). The excitation wavelengths that were used to study both Hoechst and CUR were 350 nm and 425 nm, respectively. And the emission wavelengths of Hoechst and CUR were 460 nm and 550 nm, respectively.

### 2.9. Mtt assays

The anticancer effects against glioma cell lines (C6) and the cytotoxicity on normal cell lines (HEB) were evaluated by MTT assays. Briefly, cells were passaged and mixed into a single cell suspension using a medium containing 10% FBS. The cells were seeded into 96-well plates with approximately 10,000 cells per well in a volume of 100 μL. The outer edge wells were filled with sterile PBS. The plates were incubated at 37 °C with 5% CO_2_ until the cell monolayer covered the bottom of the well. The sterilized materials with total drug concentrations of 5 μg mL^−1^ (CUR) and 10 μg mL^−1^ (TMZ) were dispersed in culture, and incubated for 24 h at 37 °C with 5% CO_2_. Then, 10 μL of MTT solution was added to each well for 4 h of incubation. Afterwards, the culture broth was removed and 100 μL of DMSO was added to each well. Plates were shaken at a low speed for 30 min in dark environment. The absorbance values were measured using the Synergy H1 enzyme-linked immunosorbent detector (BioTek, Vermont, USA) at OD490 nm. Cell viability was calculated using Equation (2) below:

(2) $$\text{Cell}\;\text{viability}\;(\%)=\frac{\text{Absorbance}\;\text{of}\;\text{test}\;\text{cells}}{\text{Absorbance}\;\text{of}\;\text{control}}\times 100\%$$

Where ‘Absorbance of test cells’ are the OD490 values of cells treated with the experimental groups, and the ‘Absorbance of control’ refers to the OD490 values of non-treated cells in the control groups.

### 2.10. Cell cycle and apoptosis analysis

During cell cycle, phosphatidylserine may be detected on the plasma membrane’s exterior surface using Annexin V fluorescein isothiocyanate (Annexin V-FITC). This assay was combined with the analysis of plasma membrane integrity (PI probe) to determine the number of apoptotic/viable cells. C6 cells were seeded onto 12-well plates at a density of 1 × 10^6^ cells per well for 24 h. Cell cultures were then treated with control (TMZ/CUR), TMZ-NLCs, CUR-NLCs and TMZ/CUR-NLCs (2:1) (at a same drug concentration of 10 μg mL^−1^). Following 24 h of incubation, cells were harvested, by trypsinisation, and counted using the NucleoCounter cytometer. To determine viability, 3 μL of PI probe was added to each sample, and all samples were incubated at 37 °C for 15 min without light. Samples were then centrifuged at 800 g for 5 min at room temperature and washed twice with Annexin V binding buffer. Cell pellets were re-suspended in 100 μL of Annexin V binding buffer, supplemented with solution binding buffer and analyzed by CytoFLEX flow cytometry, (BECKMAN COULTER, California, USA).

For apoptosis analysis, 3 × 10^5^ cells were suspended in 100 μL of Annexin V binding buffer. For each sample, 3 μL of Annexin V-7AAD conjugate and 3 μL of FITC solution were added and incubated at 37 °C for 15 min using a heating block. Samples were then centrifuged at 800 g for 5 min at room temperature and washed twice with Annexin V binding buffer. Cell pellets were then re-suspended in 100 μL of Annexin V binding buffer, supplemented with solution binding buffer and analyzed by flow cytometry.

### 2.11. Calculation of cooperativity between dual drugs

To explore the synergistic treatment effect of TMZ/CUR-NLCs (2:1) on C6 cell line, the anticancer effects were compared among TMZ-NLCs (6.67 μg mL^−1^ of TMZ), CUR-NLCs (3.33 μg mL^−1^ of CUR) and TMZ/CUR-NLCs (2:1) (6.67 μg mL^−1^ of TMZ and 3.33 μg mL^−1^ of CUR). The combinational index (CI) of dual drugs were calculated by the ‘Highest single agent’ method described by Foucquier and Guedj (Foucquier & Guedj, 2015; Ravindranathan et al., 2018). The CI value was calculated using Equation (3) below:

(3) $$\text{CI}=\frac{\left[\text{max}(E_{\text{TMZ}},\;E_{\text{CUR}})\right]}{E_{\text{TMZ}+\text{CUR}}}$$

Where ‘max (E_TMZ_, E_CUR_)’ represents the maximum effect caused by the individual agent TMZ (E_TMZ_) or CUR (E_CUR_), and ‘E_TMZ_+E_CUR_’ represents the synergistic effect caused by the two agents.

### 2.12. *In vivo* antitumor effect evaluation

BALB/c nude mice were kept at constant temperature (22–25 °C) and humidity, without a specific pathogen level barrier system. C6 cell lines (5 × 10^6^) suspended in 100 μL of PBS (pH 7.4) were injected hypodermically into the right legs of mice. The tumor volume was calculated using Equation (4) below:

(4) $$\text{Tumor}\;\text{volume}\;({\text{mm}}^{3})=\frac{\text{length}\;\times \;{\text{width}}^{2}}{2}$$

The lengths and widths of tumors were measured using a digital Vernier caliper. To evaluate the *in vivo* biodistribution of TMZ/CUR and TMZ/CUR-NLCs, mice were randomly divided into two groups (*n* = 3) and intravenously injected with samples. The doses of TMZ and CUR were 0.4 mg kg^−1^ and 0.2 mg kg^−1^, respectively. And the dose of total drugs was 0.6 mg kg^−1^. After 24 h of post-injection, the treated mice were sacrificed by overdose with pentobarbital sodium (50 mg kg^−1^), and the major organs, including heart, liver, spleen, lung, kidney and brain were dissected along with tumors for fluorescence imaging using IVIS Lumina III Series (PerkinElmer, Massachusetts, USA). CUR was used as a fluorescence dye at an excitation and emission wavelength of 430 nm and 509 nm, respectively. In order to eliminate the interference of autofluorescence from blood and other organs, the experimental mice were euthanized and perfused with 0.9% saline to flush out blood after administration. And the relevant tissues were collected and washed with PBS as well (Kang et al., 2016).

For synergistic antitumor effect evaluation, tumor-bearing mice were prepared by inoculating 5 × 10^6^ of C6 cells on the back of BALB/c nude female mice, which were randomly divided into six groups when tumor volume approached 100 mm^3^. To evaluate antitumor efficacy, tumor-bearing mice were peritumoral injected with PBS (control), free CUR/TMZ, blank-NLCs, CUR-NLCs, TMZ-NLCs and TMZ/CUR-NLCs (2:1). The doses of TMZ and CUR were 0.4 mg kg^−1^ and 0.2 mg kg^−1^, respectively. The body weights and tumor volumes of treated mice were measured every 3 days for 15 days. Tumor volume was calculated using Equation (4). After being photographed on day 15, all the treated mice were euthanised by overdose with pentobarbital sodium (50 mg kg^−1^), the major organs (heart, liver, spleen, lung, kidney and brain) and tumors were harvested. Tumors of treated mice were photographed and weighed. The excised main organs and tumors were washed with PBS and fixed in 4% formalin. The fixed organs and tumors were embedded in paraffin, sectioned and subjected to Hematoxylin and Eosin (H&E) staining. The samples were analyzed with the Leica CS2 digital pathology slide scanner (Leica, Weztlar, Germany). All the animal experiments were reviewed and approved by Animal Care and Use Committee, Guangzhou Medical University (No. 2018089).

### 2.13. Statistical analysis

Each experiment was performed in triplicates, and all data are presented as mean ± SD. Statistical analysis was performed using one-way analysis of variance with the level of statistical significance set at *p* < .05.

## 3. Results and discussion

### 3.1. Morphology, particle size and encapsulation efficiency

**Figure 1.**
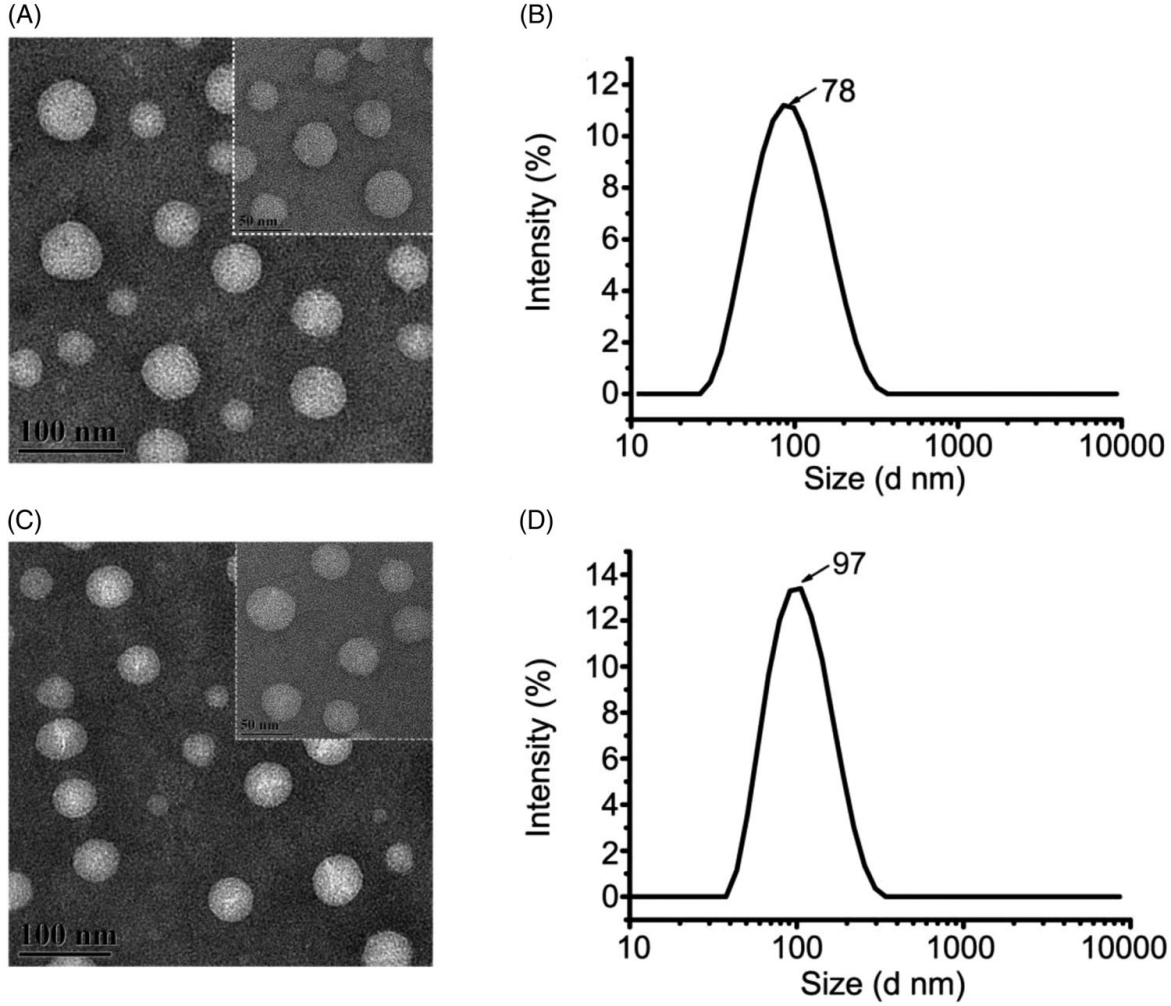
(A) TEM image and (B) particle size distribution of TMZ/CUR-NLCs (2:1). (C) TEM image of and (D) particle size distribution of blank-NLCs.

The morphologies of TMZ/CUR-NLCs (2:1) and blank NLCs were observed by TEM (Figure 1(A,C)). The obtained TMZ/CUR-NLCs (2:1) were in spherical shapes, and no aggregation was observed, demonstrating that TMZ/CUR-NLCs (2:1) could disperse well in an aqueous solution. The prepared TMZ/CUR-NLCs (2:1) were oil/water microemulsion types, and the optimum particle size was 78.49 nm ± 0.38 (Figure 1(B)), which was even smaller than blank-NLCs (97.53 nm ± 0.56) (Figure 1(D)), which may be due to the presence of the loaded drug dissolved in lipids (Gokce et al., 2012; Jiang et al., 2016; Sun et al., 2019b; Ma et al., 2020). In the pre-experiments, different weight ratio of dual drugs TMZ/CUR in NLCs were prepared (1:2, 1:1 and 2:1). We found that TMZ/CUR-NLCs (2:1) showed the smallest particle size (78.49 nm ± 0.38) than TMZ/CUR-NLCs (1:2) (83.29 nm ± 0.74) and TMZ/CUR-NLCs (1:1) (91.13 nm ± 1.08) in the pre-experiments, and the smaller particle size is preferable for crossing BBB. Therefore, we chose TMZ/CUR-NLCs (2:1) for the rest studies. The PDI and the zeta potential of TMZ/CUR-NLCs (2:1) was 0.22 ± 0.01 and −8.54 ± 0.51 mV, respectively (Supplementary Table S1). The EE of TMZ and CUR in TMZ/CUR-NLCs (2:1) was 70.90% ± 0.06 and 68.17% ± 0.20, respectively (Supplementary Table S2).

### 3.2. Thermogram properties

DSC thermograms are shown in Supplementary Figure S1. A strong absorption peak appears at 205 °C, indicating pristine TMZ was in a crystal state **(**Supplementary Figure S1(A,C)). However, the absorption peaks also appeared at 52.5 °C and 65 °C after TMZ incorporation into NLCs, possibly suggesting the transition to an amorphous state. Similarly, blank-NLCs had absorption peaks at 52.5 °C and 60 °C (Supplementary Figure S1(D)). For free CUR, there was a strong absorption peak at 180 °C; after encapsulation into NLCs, the absorption peaks showed at 50 °C and 60 °C instead (Supplementary Figure S1(B)). There were absorption peaks at 175 °C and 207.5 °C when free TMZ and CUR were physically mixed; however, these two peaks disappeared in TMZ/CUR-NLCs group (Supplementary Figure S1(C,D)). Additionally, all TMZ/CUR-NLCs groups had the same absorption peaks at 52.5 °C (Supplementary Figure S1(D)). However, they presented different absorption peaks at 62.5, 65.0, and 60.0 °C along with different intensities. The results demonstrate that different ratios of the two drugs may have affected the phase change (melting point) of lipid mixture during the DSC process, further suggesting interactions may occur between the lipids and the incorporated bioactive substances.

The following conclusions could be made from the DSC thermogram data: i) the sharp melting endothermic peaks of free drugs indicate their crystalline states. However, reduced absorption peaks were obtained after free drugs entrapment into NLCs carriers, suggesting the incorporated drugs transferred to amorphous states and new solid crystals within the lipid material were formed (Dilnawaz & Sahoo, 2013; Attama et al., 2007). ii) Smaller particle sizes and interactions between the surfactant and lipids led to a decrease in the melting enthalpy (Islan et al., 2016).

### 3.3. *In vitro* cumulative drug release study

**Figure 2.**
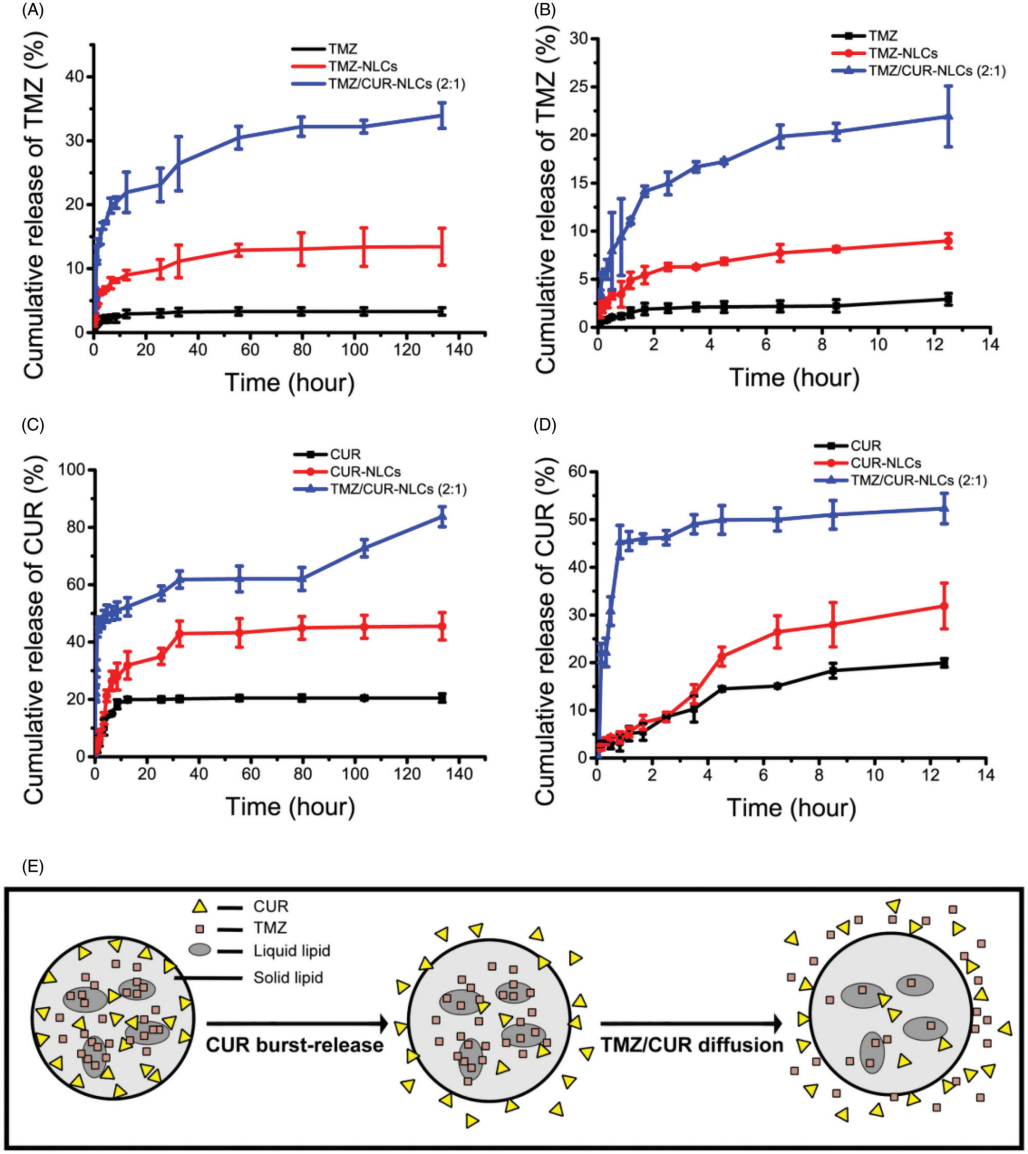
(A*) In vitro* drug release profiles of TMZ within 140 h and (B) 12.5 h. (C) *In vitro* drug release profiles of CUR within 140 h and (D) 12.5 h. (E) Proposed mechanism of sequential drug release.

The release rate of encapsulated TMZ was significantly improved after incorporating into NLCs compared to free TMZ (Figure 2(A,B)). TMZ/CUR-NLCs (2:1) showed the highest cumulative release amount of encapsulated TMZ. Moreover, the drug release rate of all dual drugs loaded NLCs groups was higher than single drug loaded NLCs groups. Similarly, NLCs nanocarriers can significantly increase the release rate of encapsulated CUR (Figure 2(C,D)); TMZ/CUR-NLCs (2:1) showed higher cumulative CUR release (∼80%) compared to CUR-NLCs (∼40%) within 140 h (Figure 2(C,D)). Interestingly, the release rate of encapsulated CUR was much higher than encapsulated TMZ. Specifically, the release rate of encapsulated TMZ was significantly slower than encapsulated CUR after 12.5 h (Figure 2(B,D)), which revealed that the incorporated drugs may be released sequentially. Possible explanation may be that CUR is more lipophilic than TMZ, so CUR was located nearby the outer lipid membranes and lipid phases of NLCs, leading to a faster diffusion rate of CUR (Figure 2(E)) than TMZ.

As seen in Supplementary Table S3, the drug release profile of TMZ/CUR-NLCs (2:1) fitted with Korsmeyer-Peppas drug release model, and the n was less than 0.45. Therefore, the drug release mechanism of TMZ/CUR-NLCs (2:1) can be explained by Fick's diffusion (Zhang et al., 2010; Shi et al., 2010). In particular, the encapsulated drugs were released by a diffusion way rather than polymer dissolving or degradation way. The results also validated that the incorporated drugs were in amorphous and disordered states, which is consistent with the thermodynamic results (Supplementary Figure S1).

### 3.4. Intracellular uptake efficiency

**Figure 3.**
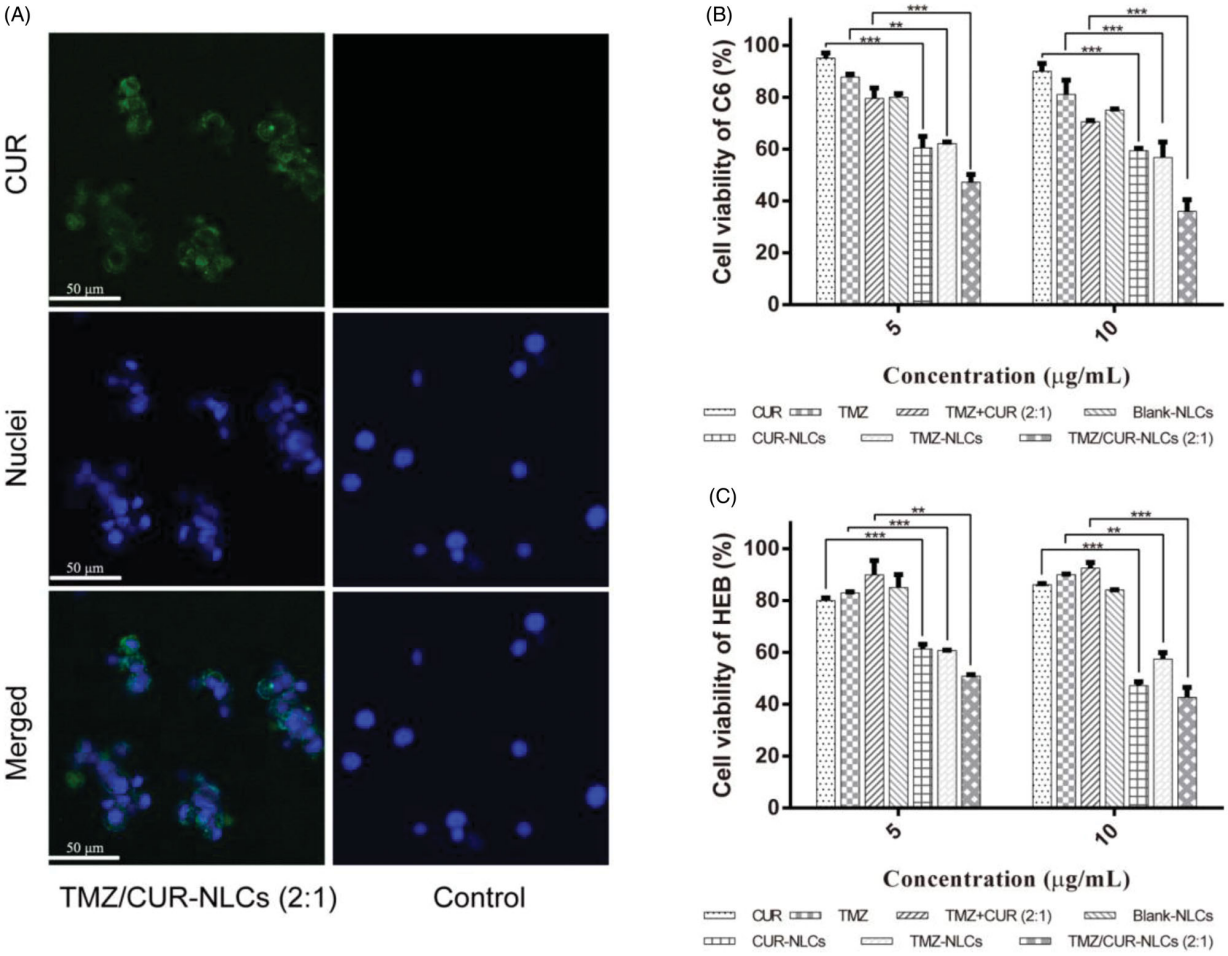
(A) Fluorescence images showing the intracellular uptake efficiency of TMZ/CUR-NLCs (2:1) and control (TMZ/CUR) after 4 h of co-cultured with C6 cells. (B) *In vitro* anticancer effect of TMZ/CUR-NLCs (2:1) on C6 cells and (C) cytotoxicity of TMZ/CUR-NLCs (2:1) on HEB cells, respectively. Scale bar: 50 μm. The results are shown in mean ± SD, *n* = 3. The statistical significance is expressed as ***p < .001, **p < .01.

Fluorescence images of C6 cell lines treated with TMZ/CUR-NLCs (2:1) and TMZ/CUR (control) for 0.5 h and 4 h were observed (Supplementary Figure S2 and Figure 3(A)). For 0.5 h of treatment, there was no obvious green fluorescence can be seen, suggesting that the incorporated drugs did not transport into the cancer cells at this time point. After 4 h of treatment, TMZ/CUR-NLCs (2:1) showed obvious green fluorescence. Moreover, green fluorescence did not appear in the control group. In summary, TMZ/CUR-NLCs (2:1) could transport into glioma cells (C6) in a time-dependent manner.

### 3.5. *In vitro* synergistic anticancer effect

As shown in Figure 3(B), CUR-NLCs had a stronger inhibitory effect on glioma cells (C6) than free CUR at concentrations from 5 to 10 μg mL^−1^. Similarly, compared with free TMZ, TMZ-NLCs showed enhanced the inhibitory effects on glioma cells. Furthermore, no inhibitory effect was observed when free CUR and TMZ were physically mixed. However, after co-loading onto NLCs, TMZ/CUR-NLCs (2:1) showed the strongest inhibitory effect than all other groups, indicating TMZ/CUR-NLCs (2:1) may have a synergistic inhibitory effect against glioma cells. The presence of CUR improved the sensitivity of C6 cells to TMZ. In addition, TMZ/CUR-NLCs (2:1) showed the most effective synergistic anticancer effect than all other groups, which is likely due to the higher TMZ content and the smallest particle size (∼ 78 nm).

The intracellular uptake and MTT results showed that TMZ/CUR-NLCs (2:1) had a higher intracellular transportation efficiency and a synergistic anticancer effect against glioma. The underlying mechanism may be due to: (i) The phase change (melting point) of the lipid mixture being affected by the different ratios of the two drugs, indicating an interaction may occur between the lipids and the bioactive substances, leading to a smaller particle size and higher solubility. (ii) Drugs were released sequentially from TMZ/CUR-NLCs (2:1). The cumulative release amount of encapsulated CUR was much higher than encapsulated TMZ within 140 h. Previous studies also demonstrated that CUR can enhance sensitivity of cancer cells to chemotherapeutic drugs, thereby improving their anticancer effects (Lu et al., 2012).

### 3.6. Cytotoxicity

The cell viability of normal human brain glial cell line (HEB) were used to evaluate the cytotoxicity of TMZ/CUR-NLCs (2:1) (Figure 3(C)). TMZ-NLCs, CUR-NLCs and TMZ/CUR-NLCs (2:1) showed inhibitory effects (∼ 50%) on HEB at concentrations of total drugs higher than 5 μg mL^−1^. However, there was no significant difference between single drug loaded NLCs and co-loaded NLCs observed. Additionally, because TMZ-NLCs and TMZ/CUR-NLCs (2:1) exhibited a similar inhibitory effect on HEB, it is likely that TMZ/CUR-NLCs (2:1) have little synergistic effect on normal cells. Furthermore, the cytotoxicity of TMZ/CUR-NLCs (2:1) increased slightly when the concentration approached 10 μg mL^−1^. More importantly, the cell viability of HEB cells was higher than C6 cells when treated with TMZ/CUR-NLCs (2:1) at a therapeutic concentration of 10 μg mL^−1^. Taken together, these results imply that TMZ/CUR-NLCs (2:1) had reduced cytotoxicity on normal cells at their suitable therapeutic dosages on cancer cells, which is owing to their controlled drug release properties.

### 3.7. Cell cycle and apoptosis measurement

**Figure 4.**
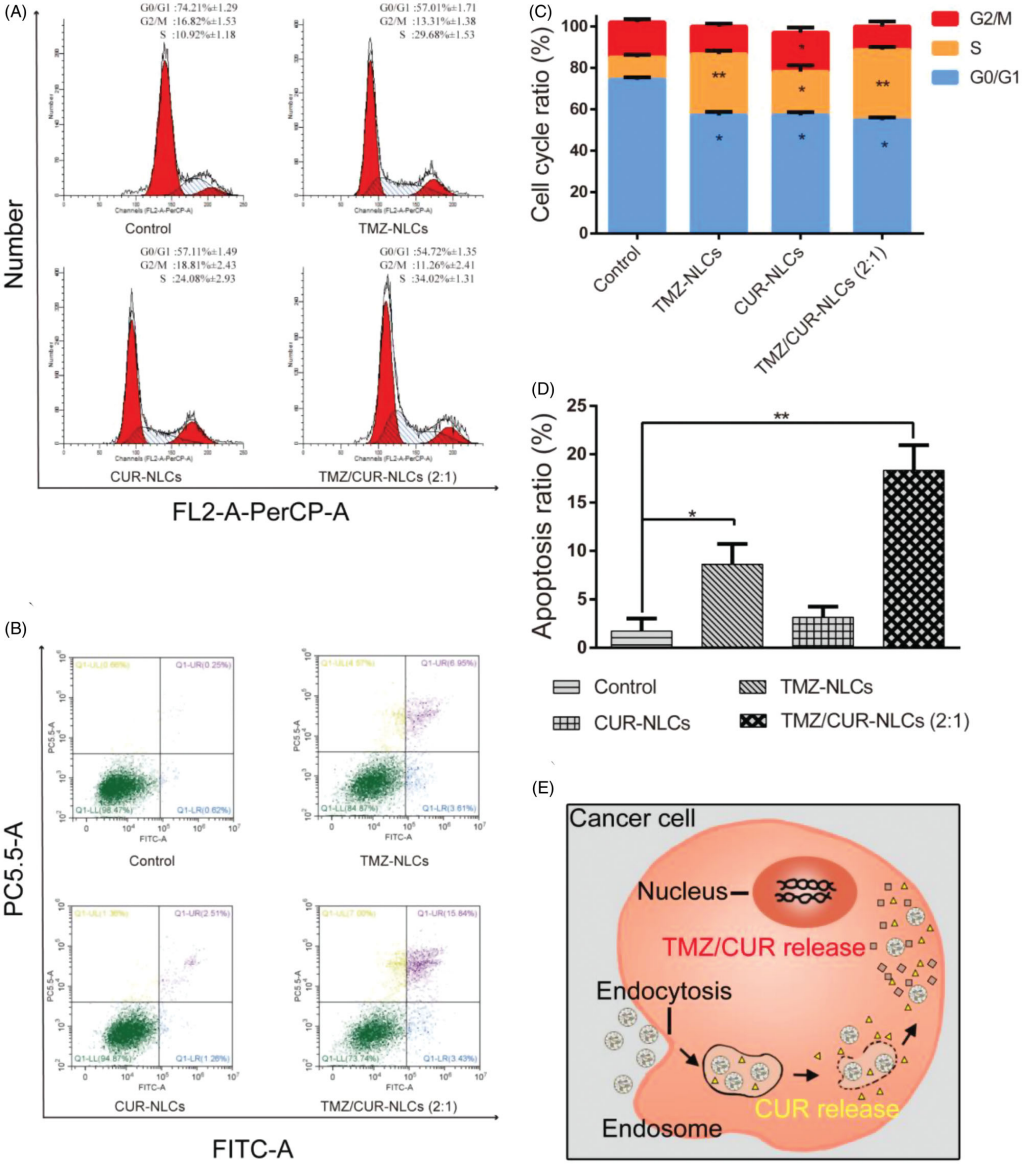
(A) Qualitative cell cycle progress and (C) quantitative cell cycle analysis of C6 cell lines treated with different samples. (B, D) Qualitative and quantitative apoptotic progression of C6 cell lines treated with control (TMZ/CUR), TMZ-NLCs, CUR-NLCs and TMZ/CUR-NLCs (2:1). (E) Illustration shows the proposed mechanism behind the synergistic anticancer effect of TMZ/CUR-NLCs (2:1) against glioma cells. The statistical significance is expressed as **p < .01, *p < .05.

To study the synergistic inhibition mechanism of TMZ/CUR-NLCs (2:1), the cell cycle progression of C6 cell lines was investigated (Figure 4(A,C)). The results showed a high percentage of cells in S phase and a lower percentage of cells in G0/G1 phase after treatment with TMZ/CUR-NLCs (2:1). Specifically, after treatment with TMZ/CUR-NLCs (2:1) the percentage of S phase cells increased to 34.02%, suggesting that TMZ/CUR-NLCs (2:1) could cause significant cell cycle arrest at the S phase.

To study the potential to induce apoptosis, C6 cell lines were treated with the control, TMZ-NLCs, CUR-NLCs and TMZ/CUR-NLCs (2:1). The results showed that TMZ/CUR-NLCs (2:1) induced the highest apoptosis rate than all other groups (Figure 4(B,D)). Specifically, the apoptotic rates of the control, TMZ-NLCs, CUR-NLCs and TMZ/CUR-NLCs (2:1) were 1.73% ± 1.3, 8.62% ± 2.1, 3.15% ± 1.1, and 18.34% ± 2.6, respectively. The results indicated that TMZ/CUR-NLCs (2:1) had a synergistic inhibition effect on C6 cell lines by inducing cell apoptosis.

Based on the results and observation, we proposed a possible synergistic anticancer mechanism of TMZ/CUR-NLCs (2:1) against glioma, which was explained by a quick release of encapsulated CUR that increased the sensitivity of C6 cell lines to encapsulated TMZ (Figure 4(E)). Afterwards, TMZ and CUR were diffused continuously and further inhibited the growth of cancer cells by inducing S phase cell cycle arrest and apoptosis.

### 3.8. Cooperativity

To confirm the synergistic effect of TMZ/CUR-NLCs (2:1), CI value was calculated using the ‘Highest single agent’ method (Foucquier & Guedj, 2015; Ravindranathan et al., 2018). The CI value of TMZ/CUR-NLCs (2:1) was 0.64 (Supplementary Figure S3), confirming the exist of synergism of TMZ/CUR-NLCs (2:1). We compared the cooperativity of TMZ/CUR-NLCs (2:1) with other nanocarriers in Supplementary Table S4. The CI value of TMZ/CUR-NLCs (2:1) was less than 1, indicating that NLCs could improve the synergistic anticancer effects of encapsulated dual drugs (Chen et al., 2018b; Wang et al., 2019; Xu et al., 2016; Zhu et al., 2017; Ali et al., 2020).

### 3.9. *In vivo* synergistic antitumor effect

**Figure 5.**
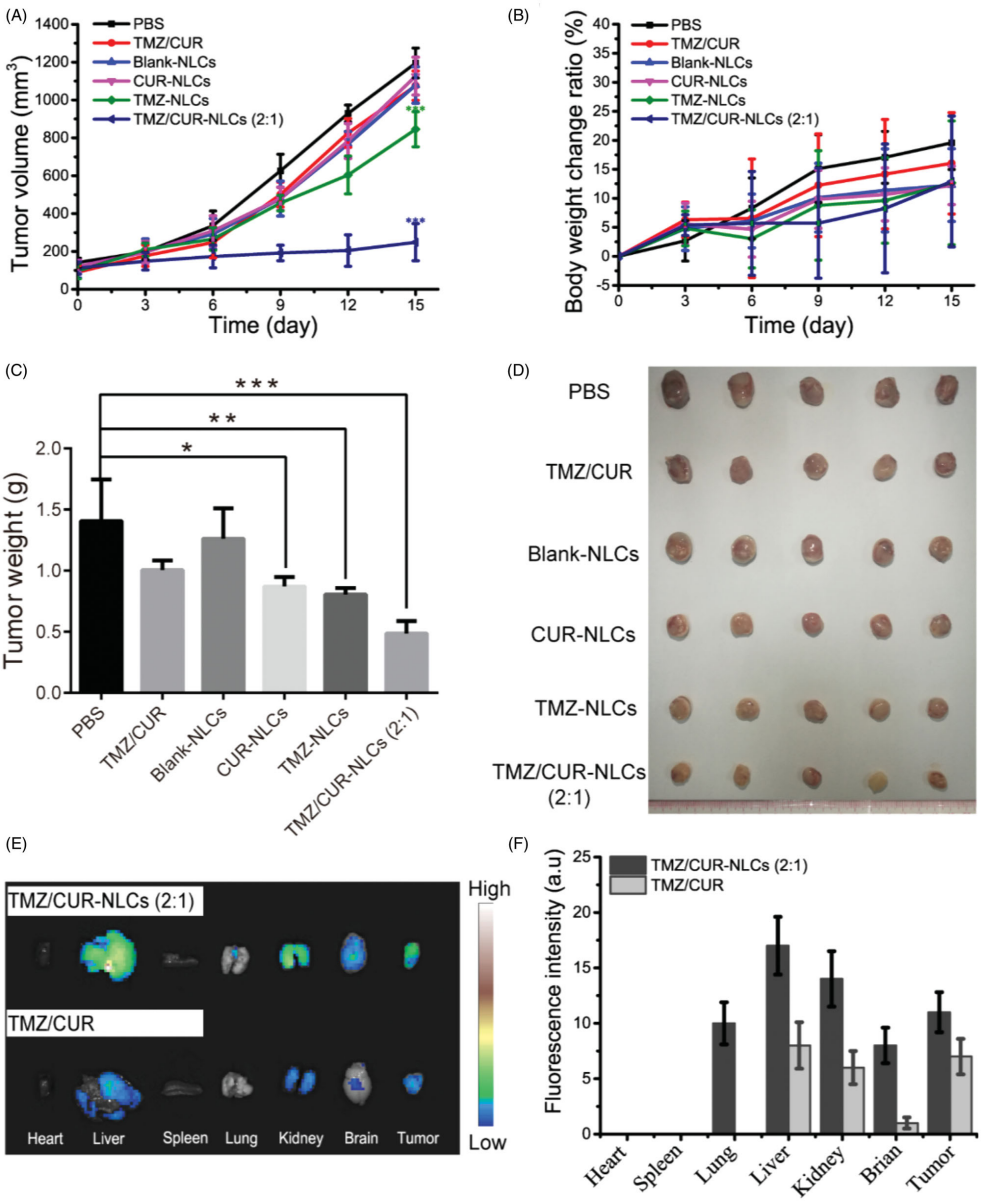
(A) The tumor volume, (B) body weight change ratio and (C) the tumor weight of mice after treatment with different samples. (D) Optical images showing tumors from treated mice. (E) *In vivo* distribution of TMZ/CUR-NLCs (2:1) and TMZ/CUR at 24 h. (F) The fluorescence intensity of TMZ/CUR-NLCs (2:1) and TMZ/CUR in different organs. The statistical significance is expressed as ***p < .001, **p < .01, *p < .05.

To further validate the *in vivo* antitumor effect of TMZ/CUR-NLCs (2:1), the glioma bearing mice were treated with different samples. The tumor growth curves showed that the tumor growth rapidly in the control group and the free CUR/TMZ group (Figure 5(A)), implying that free CUR/TMZ had no significant inhibitory effect on the growth of tumor. Tumor growth rates of mice treated with CUR-NLCs and TMZ-NLCs were lower than the free CUR/TMZ group, whereas TMZ/CUR-NLCs (2:1) showed a significant higher antitumor effect than all other groups. The body weight curves showed no significant difference among all groups (Figure 5(B)), indicating NLCs nanocarriers had good biocompatibility. Figure 5(C,D) showed that the tumor weight and optical images of separated tumors from the treated mice, confirming that TMZ/CUR-NLCs (2:1) had an enhanced synergistic treatment effect on glioma *in vivo*.

### 3.10. *In vivo* biodistribution and toxicity

The *in vivo* biodistribution of TMZ/CUR-NLCs (2:1) was studied in Figure 5(E,F). TMZ/CUR-NLCs (2:1) were injected into tumor-bearing mice through tail veins, and the biodistribution of TMZ/CUR-NLCs (2:1) in treated mice was monitored. At 24 h of post-injection, the fluorescence images showed that TMZ/CUR-NLCs (2:1) accumulated in the liver and kidney, indicating that TMZ/CUR-NLCs were metabolized mainly by liver and kidney. In addition, the fluorescence images showed that TMZ/CUR-NLCs (2:1) could accumulate in brain and tumor sites, demonstrating that TMZ/CUR-NLCs could cross BBB and target tumor.

**Figure 6.**
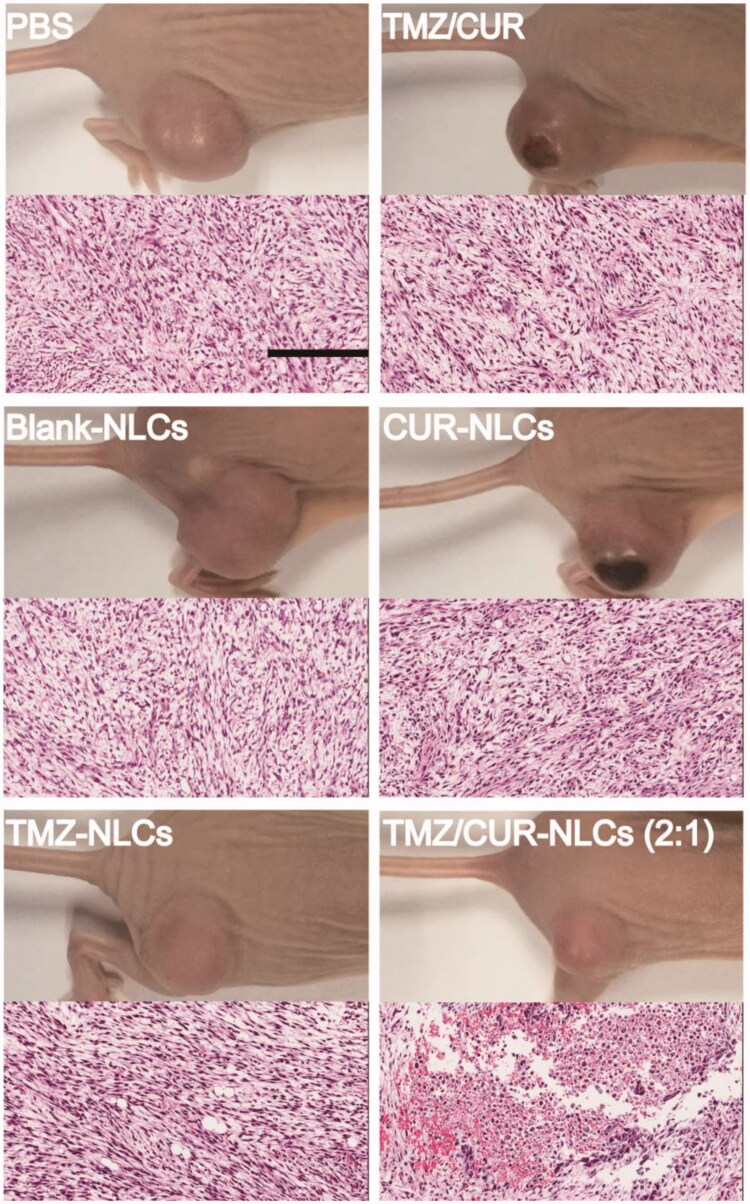
H&E staining of tumor sections separated from the treated mice. Scale bar: 200 μm.

To confirm the *in vivo* synergistic anticancer effect and investigate the potential toxicity of TMZ/CUR-NLCs (2:1), major organs (heart, liver, spleen, lung, kidney and brain) and tumor from the sacrificed mice were exercised for further pathology analysis. H&E stained images demonstrated that tumor structures were seriously damaged after treatment with TMZ/CUR-NLCs (2:1) (Figure 6). However, only slight tumor necrosis could be found in mice treated with free CUR/TMZ. There was no obvious disruption in the tumor tissue was observed of mice treated with PBS or blank-NLCs. On the other hand, compared with the PBS group, there were no obvious abnormalities or inflammatory reactions observed in the major organs of mice treated with TMZ/CUR-NLCs (2:1) (Supplementary Figure S4). In summary, these results indicated that TMZ/CUR-NLCs (2:1) were highly effective on the growth glioma due to their synergistic treatment effect and had a minimal toxicity on normal organs and tissues *in vivo*.

## 4. Conclusions

In conclusion, oil/water type TMZ/CUR-NLCs (< 100 nm) were successfully prepared in this study. Interestingly, the co-loaded drugs could be released sequentially from NLCs, and the solubility and dissolution rate of both loaded drugs were much higher than free ones. Importantly, TMZ/CUR-NLCs could accumulate at brain and tumor sites effectively, resulting in an enhanced synergistic treatment effect on the growth of glioma and a reduced toxicity on normal cells and major organs *in vitro* and *in vivo*. And the underlying mechanism of the synergistic anticancer effect of TMZ/CUR-NLCs against glioma was proposed, after being taken up by tumor cells, encapsulated CUR was burst-released first, increasing the sensitivity of the tumor cells to TMZ. Then, encapsulated CUR and TMZ were continuously released from NLCs, performing a synergistic tumor treatment effect. These findings support the safe usage of TMZ/CUR-NLCs for further clinical investigation. Therefore, NLCs are promising nanocarriers for improving synergistic tumor treatment effect, *in vitro* and *in vivo,* by sequentially delivering of dual chemotherapeutic drugs.

## Supplementary Material

Supplemental Material
