# Supplementary material for: Sequential delivery of dual drugs with nanostructured lipid carriers for improving synergistic tumor treatment effect
Source: Drug Deliv. 2020 Jul 2;27(1):983–95. doi: 10.1080/10717544.2020.1785581 (PMC8216445; doi:10.1080/10717544.2020.1785581)
Supplement: Supplemental Material [file IDRD_A_1785581_SM2768.docx]

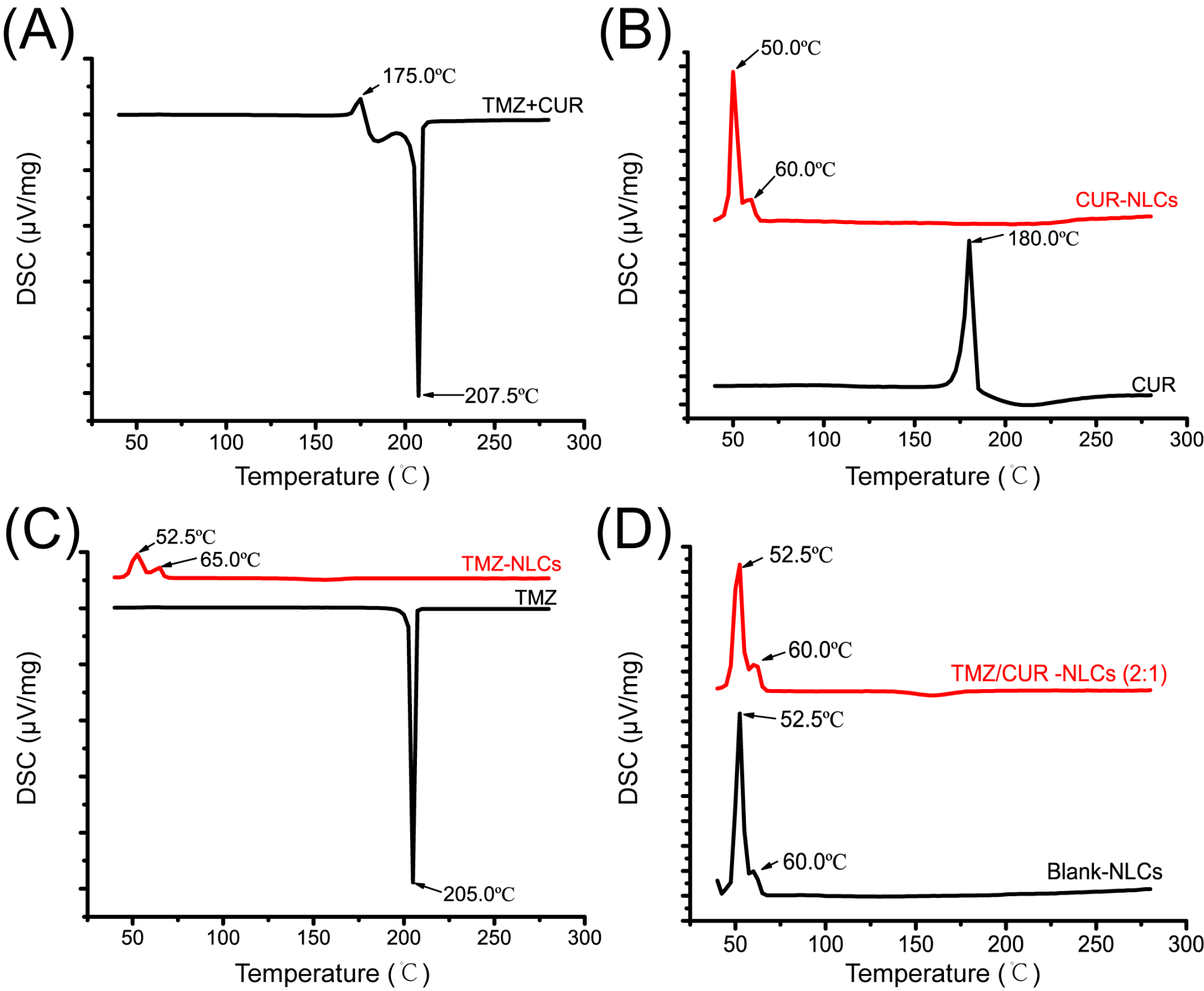


**Figure S1.** Differential scanning calorimetry of different samples.

**
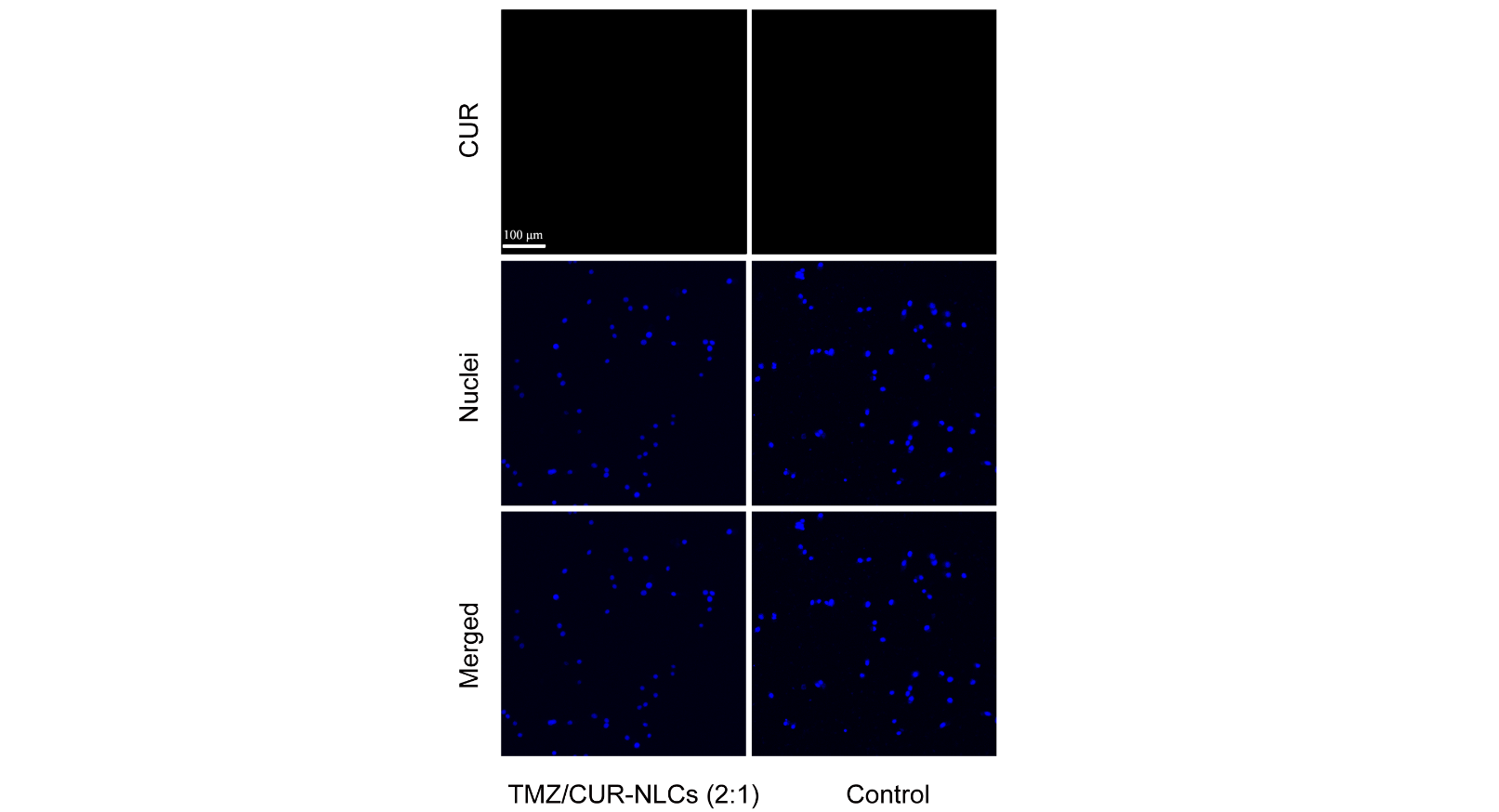
**

**Figure S2.** Fluorescence images showing intracellular uptake efficiency of TMZ/CUR-NLCs (2:1) and control (TMZ/CUR) after 0.5 h of co-cultured with C6 cells. Scale bar:100 μm.





**Figure S3.** Cooperativity of TMZ and CUR. The concentration of TMZ in TMZ-NLCs was 6.67 μg ml^-1^; the concentration of CUR in CUR-NLCs was 3.33 μg ml^-1^; the concentration of TMZ and CUR in TMZ/CUR-NLCs (2:1) was 6.67 μg ml^-1^ and 3.33 μg ml^-1^, respectively.


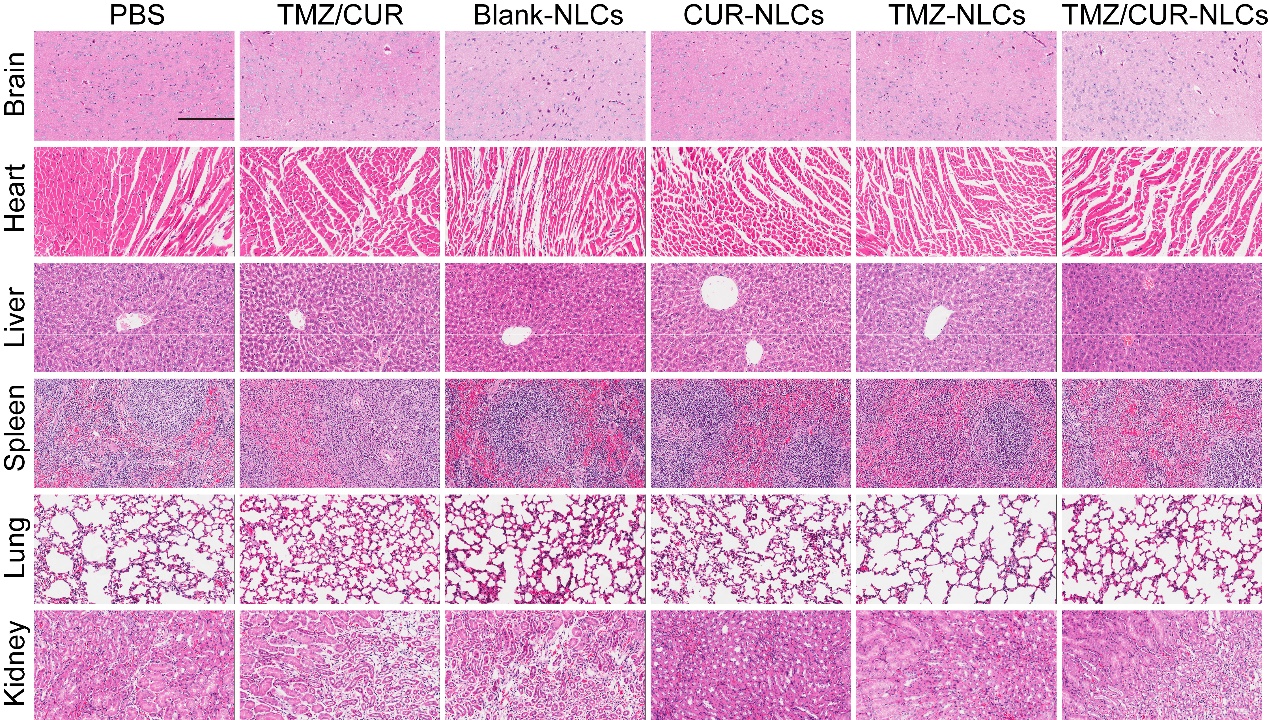
 **Figure S4.** H&E staining of brain, heart, liver, spleen, lung and kidney of mice after different treatments. Scale bar: 200 μm.

**Table S1.** Size, PDI and Zeta potential of each formulation

| Formulation | Size (nm) | PDI | Zeta potential (mV) |
| --- | --- | --- | --- |
| Blank-NLCs | 97.53±0.56 | 0.17±0.01 | -17.23±1.68 |
| CUR-NLCs | 107.93±1.41 | 0.19±0.01 | -10.8±0.66 |
| TMZ-NLCs | 85.26±1.02 | 0.19±0.02 | -6.26±1.31 |
| TMZ/CUR-NLCs (1:2) | 83.29±0.74 | 0.19±0.01 | -8.04±0.77 |
| TMZ/CUR-NLCs (1:1) | 91.13±1.08 | 0.16±0.01 | -5.45±0.52 |
| TMZ/CUR-NLCs (2:1) | 78.49±0.38 | 0.22±0.01 | -8.54±0.51 |

**Abbreviations:** CUR: curcumin; TMZ: temozolomide; NLCs: nanostructured lipid carriers.

**Table S2.** Encapsulation efficiency of each group

| Formulation | Encapsulation efficiency (%) | |
| --- | --- | --- |
|  | CUR | TMZ |
| CUR-NLCs | 85.58±0.33 |  |
| TMZ-NLCs |  | 90.95±0.08 |
| TMZ/CUR-NLCs (2:1) | 68.17±0.20 | 70.90±0.06 |

**Abbreviations:** CUR: curcumin; TMZ: temozolomide; NLCs: nanostructured lipid carriers.

**Table S3.** Fitting equation of reference formulation release model

| Formulation | Models | | | | | | | |
| --- | --- | --- | --- | --- | --- | --- | --- | --- |
|  | Zero-order | R^2^ | Weibull | R^2^ | Higuchi | R^2^ | Korsmeyer-Peppas | R^2^ |
| **Release of CUR** |  |  |  |  |  |  |  |  |
| CUR-NLCs | F=13.3608+0.3430*t | 0.6012 | F=100*{1-Exp[-(t^0.3980)/9.3000]} | 0.9153 | F=5.8187+4.4487*t^0.5 | 0.8255 | F=-34.3243+43.6980*t^0.1362 | 0.9342 |
| TMZ/CUR-NLCs (2:1) | F=39.0059+0.3556*t | 0.5174 | F=100*{1-Exp[-(t^0.2380)/2.2899]} | 0.8352 | F=31.7579+4.4530*t^0.5 | 0.6612 | F=35.5429*t^0.1592 | 0.8025 |
| CUR | F=9.1624+0.1278*t | 0.4066 | F=100*{1-Exp[-(t^0.2602)/12.6330]} | 0.8139 | F=5.9527+1.7683*t^0.5 | 0.6506 | F=-875.7640+883.1432*t^0.004 | 0.8776 |
|  |  |  |  |  |  |  |  |  |
| **Release of TMZ** |  |  |  |  |  |  |  |  |
| TMZ-NLCs | F=5.3845+0.0830*t | 0.662 | F=100*{1-Exp[-(t^0.2499)/21.4206]} | 0.9722 | F=3.6316+1.0568*t^0.5 | 0.8712 | F=-9.1545+13.6667*t^0.1089 | 0.9876 |
| TMZ/CUR-NLCs (2:1) | F=13.1983+0.2033*t | 0.6711 | F=100*{1-Exp[-(t^0.2679)/8.4467]} | 0.9755 | F=8.9685+2.5703*t^0.5 | 0.8704 | F=-28.3364+39.4870*t^0.09430 | 0.9903 |
| TMZ | F=1.6976+0.0176*t | 0.4801 | F=100*{1-Exp[-(t^0.1931)/67.0919]} | 0.9011 | F=1.2780+0.2376*t^0.5 | 0.7193 | F=-1167.5042+1168.9770*t^0.0004 | 0.9638 |

**Abbreviations:** CUR: curcumin; TMZ: temozolomide; NLCs: nanostructured lipid carriers.

**Table S4.** Dual drugs systems of tumor treatment

| Drug 1 | Drug 2 | Materials | CI | Refs |
| --- | --- | --- | --- | --- |
| MTX | PMX | CNPs | 0.28 | Chen et al., 2018b |
| DOX | PDA | DOX-PDA-gossypol NPs | 0.23 | Wang et al., 2019 |
| DOX | AXI | MC | 0.44 | Xu et al., 2016 |
| VCR | QU | LPNs | 0.25 | Zhu et al., 2017 |
| PMT | EA | MSNPs | 0.89 | Ali et al., 2020 |

**Abbreviations:** CI: combination index; MTX: methotrexate; PMX: pemetrexed; CNPs: chitosan nanoparticles; DOX: doxorubicin; PDA: polydopamine; AXI: axitinib; MC: micelle; VCR: vincristine; QU: quercetin; LPNs: lipid-polymeric nanocarriers; PMT; pemetrexed; EA: ellagic acid; MSNPs: mesoporous silica nanoparticles.
